# Low density neutrophils (LDN) in postoperative abdominal cavity assist the peritoneal recurrence through the production of neutrophil extracellular traps (NETs)

**DOI:** 10.1038/s41598-017-19091-2

**Published:** 2018-01-12

**Authors:** Rihito Kanamaru, Hideyuki Ohzawa, Hideyo Miyato, Shiro Matsumoto, Hidenori Haruta, Kentaro Kurashina, Shin Saito, Yoshinori Hosoya, Hironori Yamaguchi, Hiroharu Yamashita, Yasuyuki Seto, Alan Kawarai Lefor, Naohiro Sata, Joji Kitayama

**Affiliations:** 1 Department of Gastrointestinal Surgery, Jichi Medical University, Shimotsuke, Japan; 2 Department of Gastrointestinal Surgery, The University of Tokyo, Tokyo, Japan

## Abstract

Many types of immune cells appear in peritoneal cavity after abdominal surgery. In patients who underwent laparotomy due to gastric cancer, peritoneal lavages were obtained before and after surgical procedure. Cells were recovered from intermediate layer after Ficoll-Hypaque centrifugation and analyzed for phenotypes and functions, especially focused on low density neutrophils (LDN). The number of CD66b (+) LDN with mature phenotype was markedly elevated in postoperative as compared with preoperative lavages. Short term culture of the purified LDN produced many threadlike structures positive for SYTOX, nucleic acid staining, as well as histone and myeloperoxidase, suggesting the NETs formation. Human gastric cancer cells, MKN45, OCUM-1 and NUGC-4, were selectively attached on the NETs, which was totally abolished by the pretreatment of DNAse I. Intraperitoneal (IP) co-transfer of the LDN with MKN45 in nude mice strongly augments the metastasis formation on peritoneum, which was strongly suppressed by the following IP administration of DNAse I. Many NETs-like structures were detected on the surface of human omental tissue resected by gastrectomy. NETs on peritoneal surface can assist the clustering and growth of free tumor cells disseminated in abdomen. Disruption of the NETs by DNAse might be useful to prevent the peritoneal recurrence after abdominal surgery.

## Introduction

The peritoneal cavity is a common target of metastatic gastrointestinal and ovarian cancer cells, but the mechanisms leading to peritoneal metastasis have not been fully elucidated yet. In gastric cancer (GC), which is the second leading cause of cancer death and the fourth most common cancer in the world^1,2^, peritoneal metastasis frequently develops in patients during the course of the disease progression. Especially, when gastric serosa is infiltrated, approximately half of the patients developed peritoneal recurrence in spite even radical surgery^3^. Although heated intraperitoneal chemotherapy^4,3^ or the postoperative chemotherapy^5,6^ partially reduced the rate of peritoneal recurrence, the benefits of those adjuvant therapies are still limited, and thus the research for more effective treatments is mandatory.

Inflammation is described as one of the hallmarks of cancer and the interaction between neoplastic cells and immune cells in microenvironment have been implicated in various steps of tumor development and metastasis^7,8^. Peritoneal cavity contains many types of immune cells which can mediate direct contact with tumor cells expoiliated from the serosal surface of primary tumors. In general, lymphocytes and macrophages are considered to be the major leukocytes in peritoneal immunity^9,10^. In contrast, after abdominal surgery, many neutrophils appear in abdominal cavity through the direct exudate or intraoperative bleedings. However, the role of the influx of neutrophils on postoperative recurrence is still unclear.

Indeed, neutrophils are the most abundant leukocytes and well-known to work as the first line defence against pathogens. Recently, however, neutrophils have been increasingly recognized as a potential key player to regulate the various steps in tumor progression^11,12^. Previous studies have shown that neutropliles can be divided into different subpopulations, N1 and N2 neutrophils, which exert anti- and pro-tumorigenic properties, respectively, and tumor derived factors have been shown to induce the formation of N2 phenotype^13,14^. Clinical studies have shown the strong correlation between high neutrophils counts in circulating blood with poor outcome in various types of human cancers^15^. These facts inspired us to examine the function of these neutrophils resident in postoperative abdominal cavity

Under normal conditions, circulating neutrophils can be separated with mononuclear cells by Ficoll-Hypaque density gradient preparations. However, it has been shown that the frequency of the granulocytes co-purified with mononuclear cells are significantly increased in various diseases, such as autoimmune diseases^16,17^, sepsis^18^, cancer^19,20^ and HIV infection^21^, which are designated as low density neutrophils (LDN). Those studies have shown that human LDN are morphologically divided into two distinct subpopulations with different functional characteristics; mature type of garanulocytes with multilobular nuclea and relatively immature granulocytes with less segmented nucleus as compared with high density neutrophils (HDN). Although the origin and pathological roles of these LDNs have not been fully elucidated, previous studies suggest the LDN contributes to lupus pathogenesis as well as the development of organ damage through enhanced pro-inflammatory responses^22,23^. More recently, Sagev *et al*. have demonstrated that pro-tumor LDN can be induced from HDN by TGF-β stimulation in cancer bearing host, suggesting the possible involvement of LDN in tumor progression^24^.

Another important feature of LDN is the capacity to form neutrophil extracellular traps (NETs). NETs are a complex structures composed of a chromatin decorated with histons, proteases and granular and cytosolic proteins, and was recognized as an important antimicrobial mechanism to immobilize and kill the pathogens^25^. More recently, however, NETs in hepatic sinusoid have been shown to effciciently trap circulating tumor cells, which results in the augmentation of hepatic metastasis in systemic infection^26^ or ischemia/reperfusion^27^ models. Moreover, the extracellular DNA has been shown to enhance migration and invasion behabiour in murine^27^ and human^26,28^ tumor cells. In fact, NETs structures can often be detected in proximity to human tumors^27,29,30^. In addition, Demers *et al*. have shown that various tumors are capable to predispose circulating neutrophils to produce NETs causing syetemic thromosis which is often associated with human cancer^31^. All these facts strongly suggest the positive contribution of NETs on tumor progression and metastasis.

In this study, therefore, we focused on the neutrophils recovered from peritoneal lavages who received curative gastrectomy and investigated their mechanical involvement in metastsis formation in abdominal cavity.

## Results

### Postoperative peritoneal lavages contain many low density neutrophils (LDN) with mature phenotype

**Figure 1:**
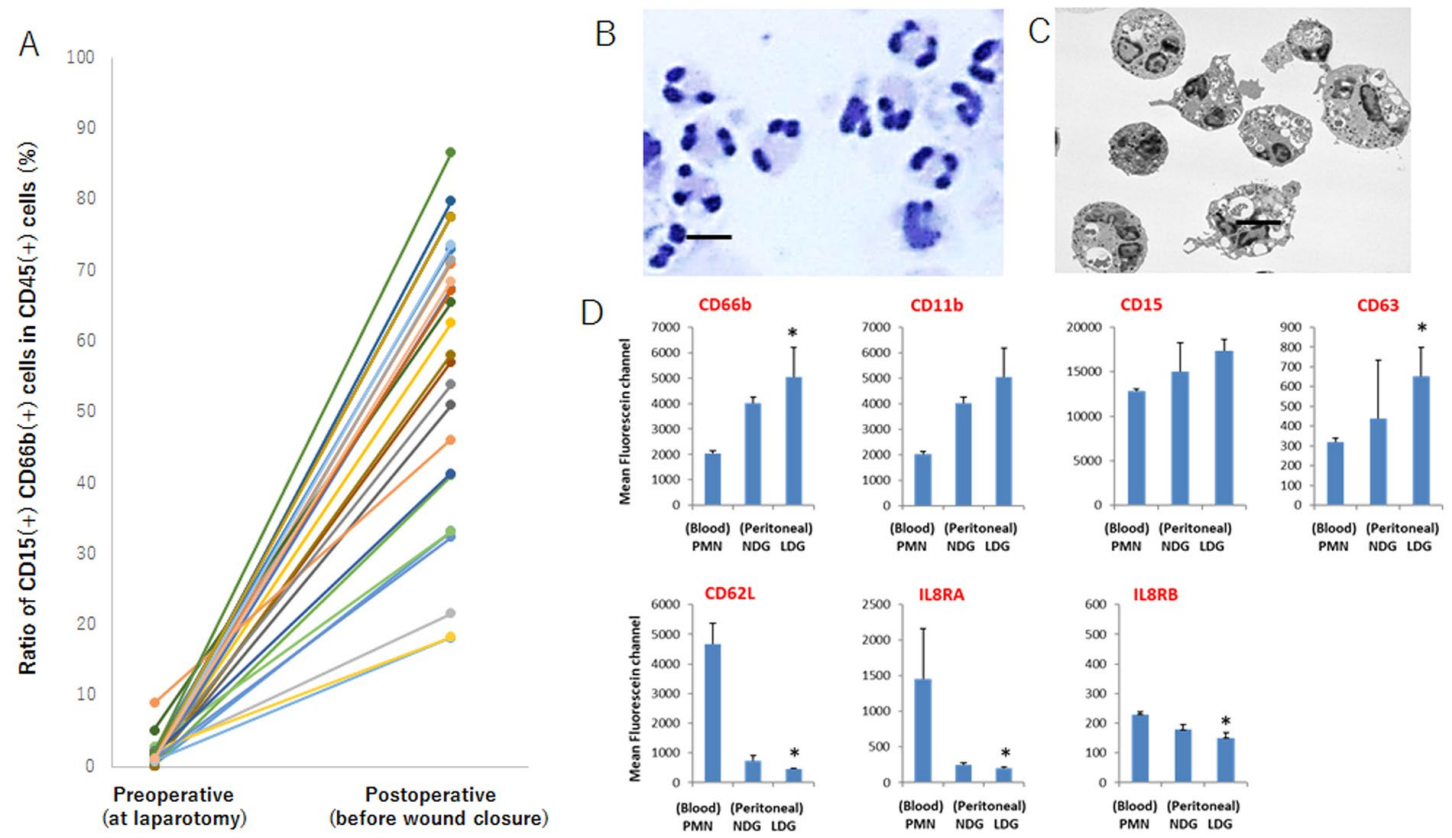
(**A**) Preoperative and postoperative peritoneal lavages were obtained at laparotomy and before wound closure, respectively. In cellular components recovered from mononuclear cell layer after Ficolll-Hypaque centrifugation, the percentages of CD15(+) CD66b(+) neutrophils in CD45(+) whole leukocytes were calculated in flow cytometry. Giemsa staining (**B**) and transmission electoron microscopy (**C**) of the postoperative low density neutrophils (LDN). Black bars show 10 μm. (**D**) Antigen expression pattern of LDN and HDN in postoperative lavage and circulating PMN. Cells obtained from 3 different patients were stained with specific mAbs and mean fluorescein channels (MFC) in granulocyte region were measured with flowcytometry. Data show mean ± SD of the 3 patients. *P value was less than 0.05

Cells were recovered from peritoneal lavages before and after operative procedure in 27 patients who received abdominal surgery by gastric cancer. None of the patients received intraperitoneal chemotherapy before or during surgery. Among the cells recovered from intermediate mononuclear cell layer by Ficoll-Hypaque centrifugation, granulocytes with CD15(+) CD66b(+) phenotype were considered as low density neutrophils (LDN) and the percentages of the LDN against CD45(+) whole leukocytes were calculated. In all cases, the ratio of LDN in preoperative lavages were minor (M = 1.14%, 0.06~8.96%), whereas markedly elevated in postoperative samples (M = 62.6%, 18.2~86.7%, p < 0.001) (Fig. 1A). The majority (89 ± 5.1%) of the LDN in postoperative samples showed multi-lobular nucleus by Giemsa staining (Fig. 1B and Supplementry Fig. 1) and contained many vacuoles under the transmission electron microscopy (Fig. 1C). Flowcytmetric analysis revealed that the mean fluorescein channels of CD11b and CD63, a degranulation marker, were significantly increased in peritoneal LDN than circulating PMN, and tended to be higher than high density neutrophils (HDN) in the same peritoneal lavages (Fig. 1D and Supplement Fig. 2). The expression levels of CD15 and CD66b showed the similar trend. In contrast, LDN showed significantly reduced expression of CD62L as well as IL-8 receptors as compared with circulating PMN. All the findings indicate that the peritoneal LDN are somehow activated mature type neutrophils which were recruited and activated by the inflammatory stimulus caused by surgical manipulation.

### LDN in postoperative lavage form massive NETs (neutrophil extracellular traps) by short term culture ***in vitro***

**Figure 2:**
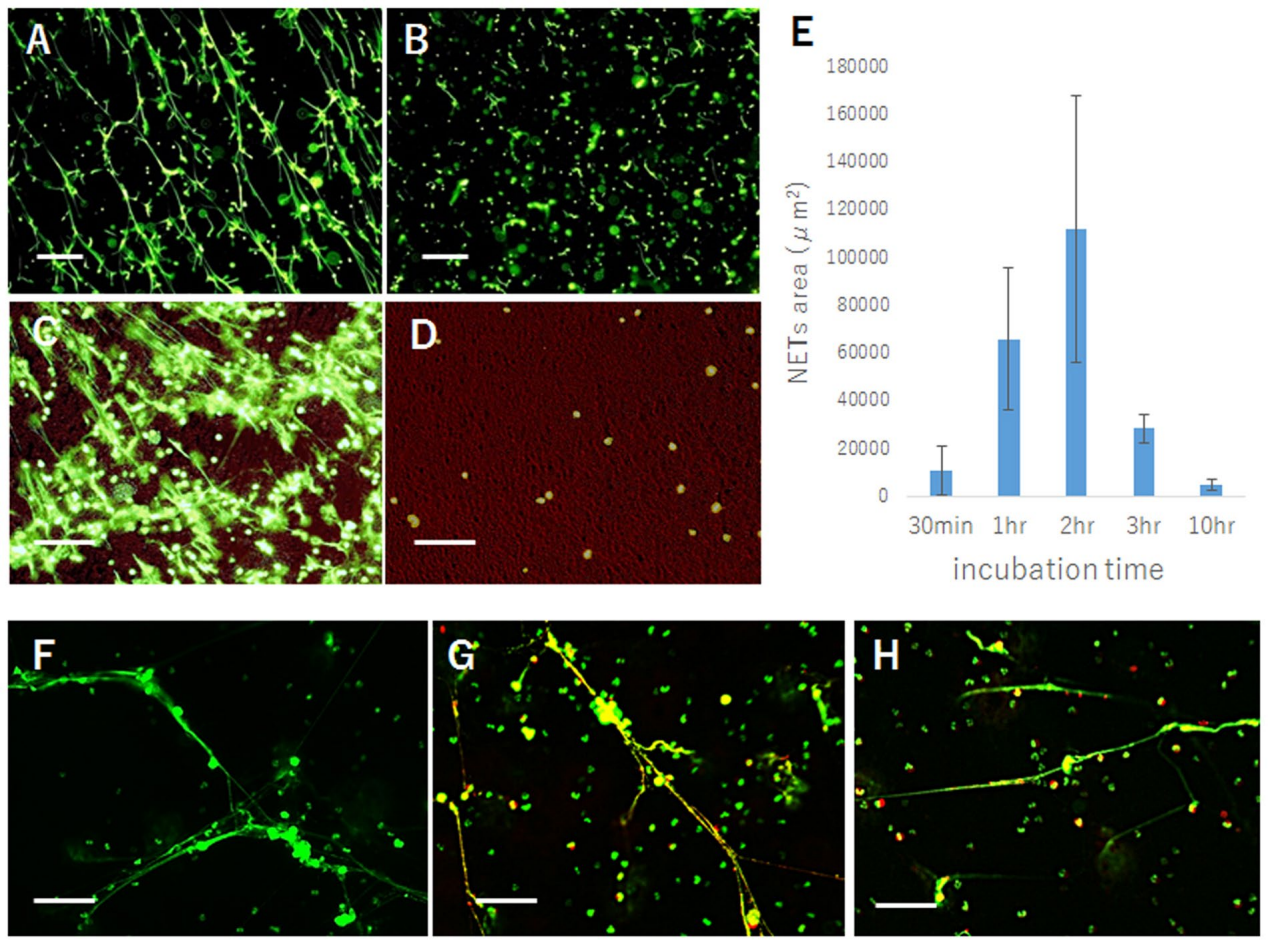
(**A**) Cells (5 × 10^6^) derived from a postoperative lavage, which contains 80% of LDN, were placed on Poly-L-lysine coated 6 well plate and cultured for 2 hours and SYTOX green was added and immediately observed under fluorescein microscopy. (**B**) the same culture was pretreated with DNAse (100 u/ml) for 5 min before the addition SYTOX green. After the magnetic separation, same number of CD66b(+) LDN (**C**) and CD66b(−) cells (**D**) were cultured for 2 hour and NETs formation was visualized by SYTOX green. (**E**) Purified LDN were cultured for indicated periods and NETs were visualized by SYTOX green. In 3 randomly selected fields, areas of the NETs structure were measured using ImageJ software. Data show mean ± SD. (**F**,**G**) Purified LDN (5 × 10^6^) cultured on 6 well plate for 2 hours and stained with SYTOX green were incubated with mAbs to myeloperoxidase (**G**), histone (**H**) as well as control mouse IgG (**F**) at the concentration of 5 μg/ml for 1 hour, washed twice and followed by PE-conjugated secondary antibody for 30 min. In each sample, two photos were taken under optical wavelength filters for FITC or PE and superimposed. White bars show 100 μm.

In samples which consisted many LDN (more than 80%), the peritoneal cells were cultured in DMEM with 10% FCS for several hours and examined for the staining of SYTOX green, membrane impermeable dye to stain nucleic acid. As shown in Fig. 2A,B, many thread-like structures were observed under the fluorescein microscope, which was totally destroyed by the pretreatment of DNAse I for 5 min. When LDN and other mononuclear cells were separated using anti-CD66b conjugated with microbeads, similar staining for SYTOX was detected massively in CD66b(+), but rarely in CD66b(−) fractions (Fig. 2C,D). This suggests that they are extracellular DNA expelled from neutrophils, which was designated as NETs (neutrophil extracellular traps). To confirm this, we performed immunostaining of the LDN culture using mAbs to myeloperoxidase or histone, which clearly demonstrates the co-localization of those molecules on SYTOX-positive fibers (Fig. 2F–H). When the NETs structure was quantified using image processing software, the LDN formed NETs within 30 min culture which reached maximum at 2 hours and then gradually decreased thereafter, probably due to the DNAse contained in serum (Fig. 2E).

### Human gastric cancer cells selectively attached to the NETs and proliferate on LDN monolayer

**Figure 3:**
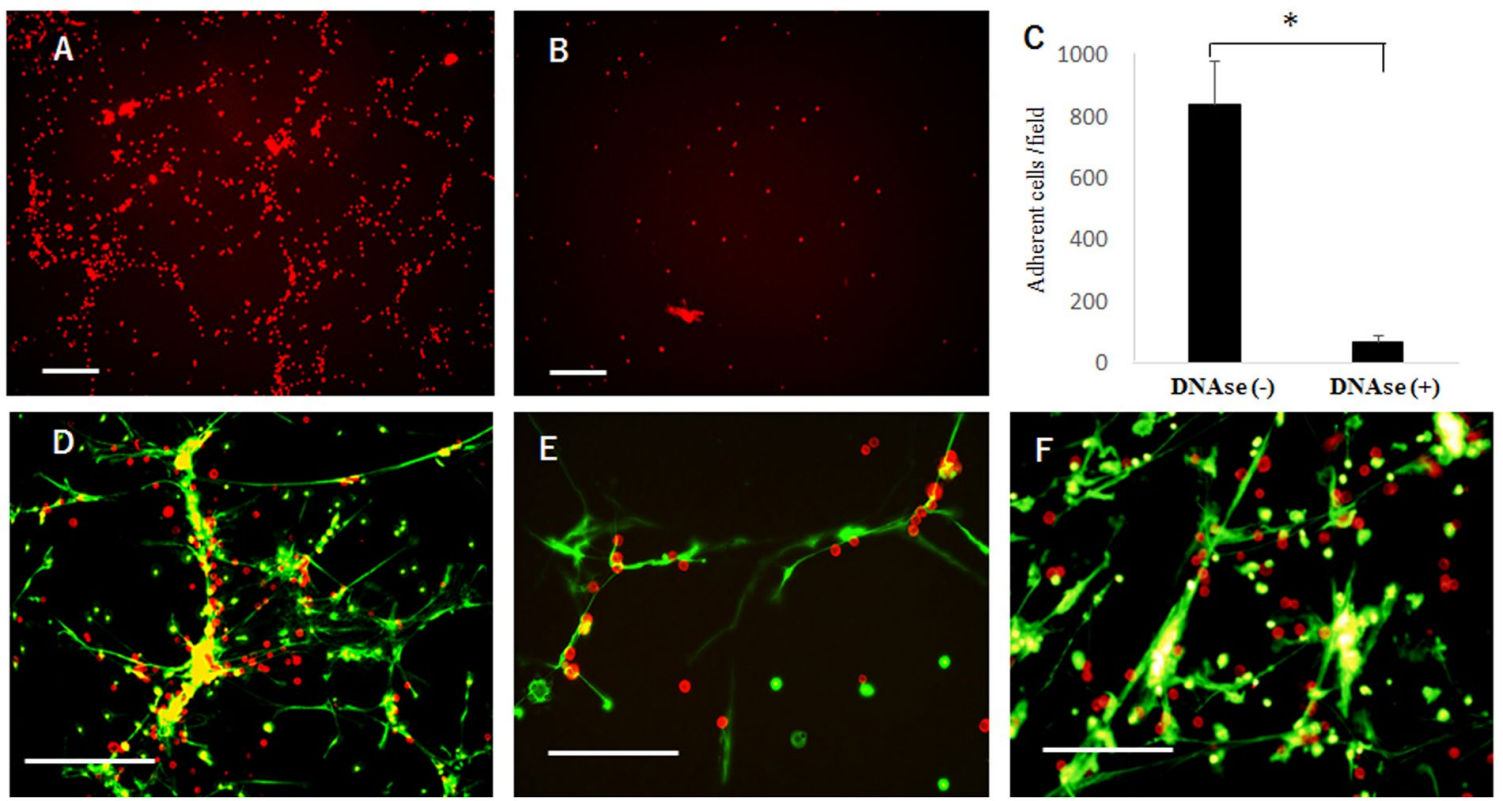
LDN (5 × 10^6^) were cultured on poly-L-lysine coated 6 well plate for 2 hours. Three different human gastric cancer cell lines, MKN45, OCUM-1 or NUGC-4, were stained red by PKH26 and 1 × 10^6^ cells suspended in 1 ml DMEM were added and incubated for 5 min. After gentle washing, the attached tumor cells were counted under the fluorescein microscope. (**B**) LDN monolayer was pretreated with 100 u/ml DNAse for 5 min just before the addition of tumor cells. (**C**) The number of the attached cells were counted at 3 randomly selected fields and mean ± SD was expressed. P value was less than 0.05 (**D**) Immediately after the adhesion experiment, SYTOX green was added and NETs structure was visualized under the optical wavelength filter for FITC, and superimposed on the photo taken under the wavelength for PKH26. The same experiments were performed for OCUM-1 (**E**) and NUGC-4 (**F**). White bars show 100 μm.

Next, we examined the adhesion behavior of human gastric cancer cell lines with the NETs. The purified LDN were cultured for 2 hours on plate and MKN45 cells labelled with PKH26 were added and co-incubated for 5 min. After the wells were gently washed, many MKN45 cells were observed to attach to the LDN layer (Fig. 3A,C). In contrast, only few cells were attached, when the LDN monolayer was pretreated with DNase I (Fig. 3B,C). Moreover, almost all the attached MKN45 were co-localized with SYTOX green positive NETs structures (Fig. 3D). Similar adhesion pattern was observed in other gastric cancer cells, OCUM-1 and NUGC-4 (Fig. 3E,F).

When the same experiment was performed on NDN culture, some MKN45 attached on NDN culture and most of the adhesive tumor cells were co-localized to SYTOX green positive NETs structure, although the number of adhesive cells as well as amount of NETs were significantly less than those on LDN (Supplement Fig. 3). This indicates that NDN in postoperative lavages can also produce tumor adhesive NETs although the capacity is much less than LDN.

**Figure 4:**
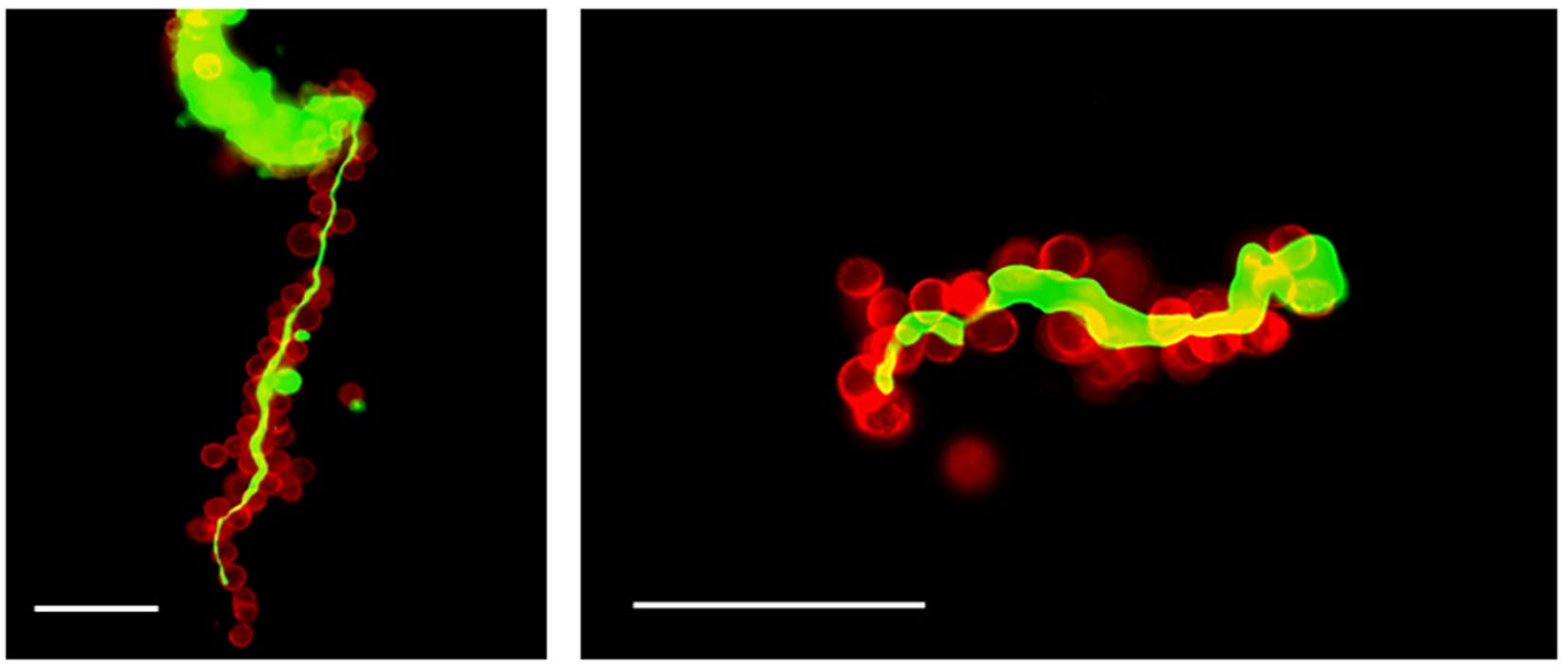
After the adhesion experiments using MKN45 (**A**) and OCUM-1(**B**) as described in Fig. 4, the wells were washed again and continued the co-culture in 10%FCS + DMEM for additional 18 hours. The photos of the parts where NETs structure remained were taken under different filters and superimposed as described in the legends of Fig. 3. White bars show 100 mm.

Since NETs have been originally described to be cytotoxic to microbes, we next examined the fate of the tumor cells attached to NETs. Interestingly, MKN45 and OCUM-1 attached to NETs started to proliferate in several hours as they did without NETs, while the NETs were gradually disappeared (Fig. 4, Supplement Video)

### Co-transfer of the peritoneal LDN enhanced peritoneal metastasis ***in vivo***

**Figure 5:**
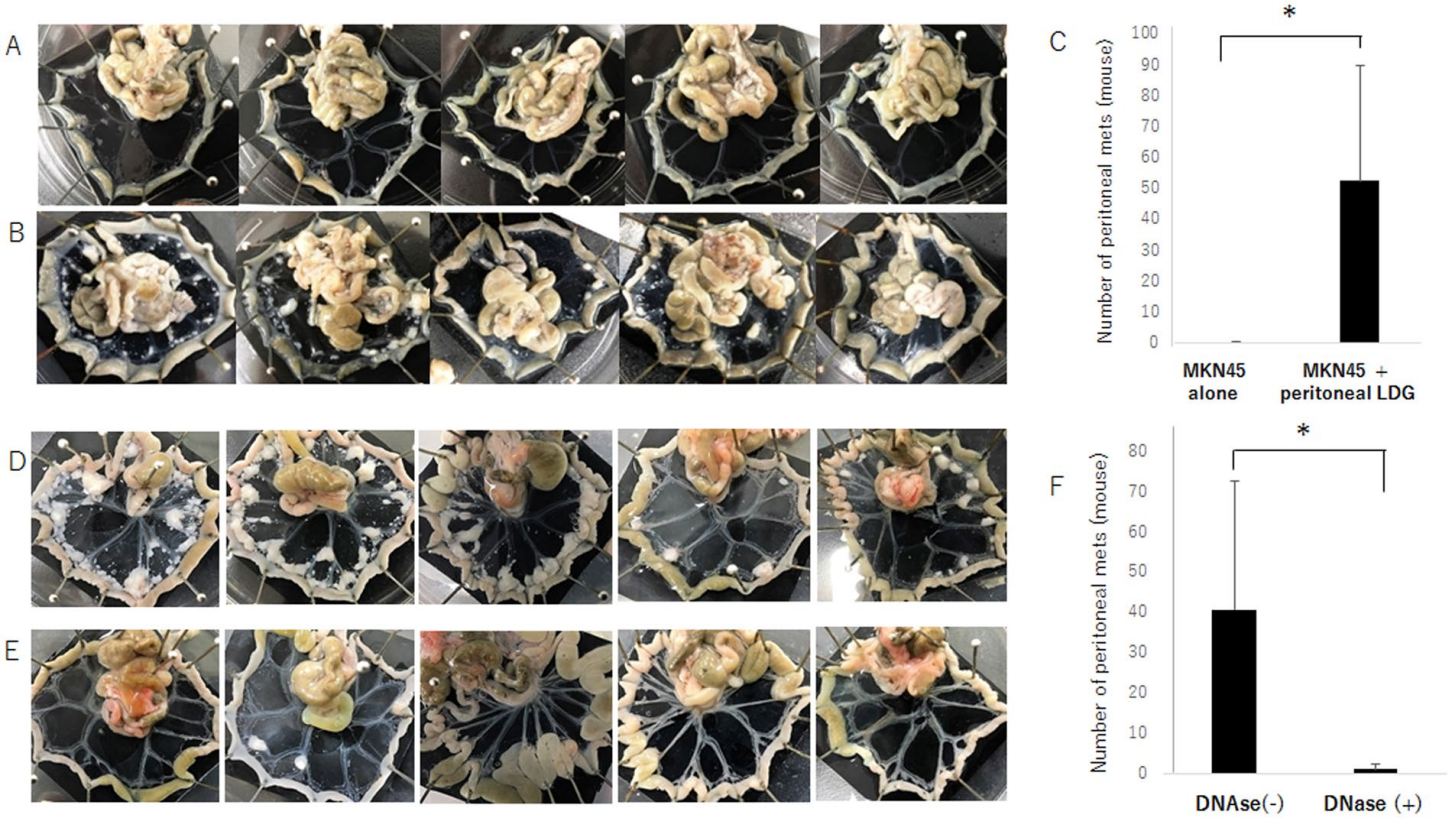
MKN45 (1 × 10^5^) with (**B**) or without (**A**) purified LDN (1 × 10^7^) suspended in 1 ml HBSS were intraperitoneally injected into the balb/c mice. After 4 weeks, the mice were sacrificed and the number of macroscopically detectable metastatic nodules on mesentery were counted. Same experiments were performed for 3 times and mean ± SD in 5 mice in a representative experiment were shown. (**C**). (**D**–**F**) Similarly, MKN45 and purified LDN were intraperitoneally co-transferred into the balb/c mice. Then, 1 ml of PBS alone (**D**) or DNAse suspended in PBS at the concentration of 1000 u/ml (**E**) were intraperitoneally administrated just after the cell injection. Four weeks later, nodules on mesentery were counted. Data show mean ± SD in 5 mice in 3 different experiments. *P value was less than 0.05.

Based on the *in vitro* results, we examined whether the presence of the LDN in abdominal cavity enhances the peritoneal metastasis in murine model. As shown in Fig. 5A,C, intraperitoneal injection of a relatively small number (1 × 10^5^) of MKN45 in balb/c nude mice produced only a small number of metastases on peritoneum in 1 of the 5 mice at day 28. However, when the same number of MKN45 together with the purified LDN (1 × 10^7^) derived from in postoperative lavage, numerous metastatic nodules were observed in all the mice (Fig. 5B,C). Co-transfer of the same number of NDN appears to slightly increase the number of peritoneal metastasis of MKN45 although the difference did not reach the statistical significance (Supplement Fig. 4). On the other hand, when DNAse I was additionally injected after the co-transfer of MKN45 and LDN, the number of metastatic nodules on peritoneum was markedly reduced (Fig. 5D–F). These facts strongly suggest that NETs on peritoneum produced by exudative LDN attach floating tumor cells and support the development of peritoneal metastasis.

### NETs like structures exists on surgically removed human omental tissue

**Figure 6:**
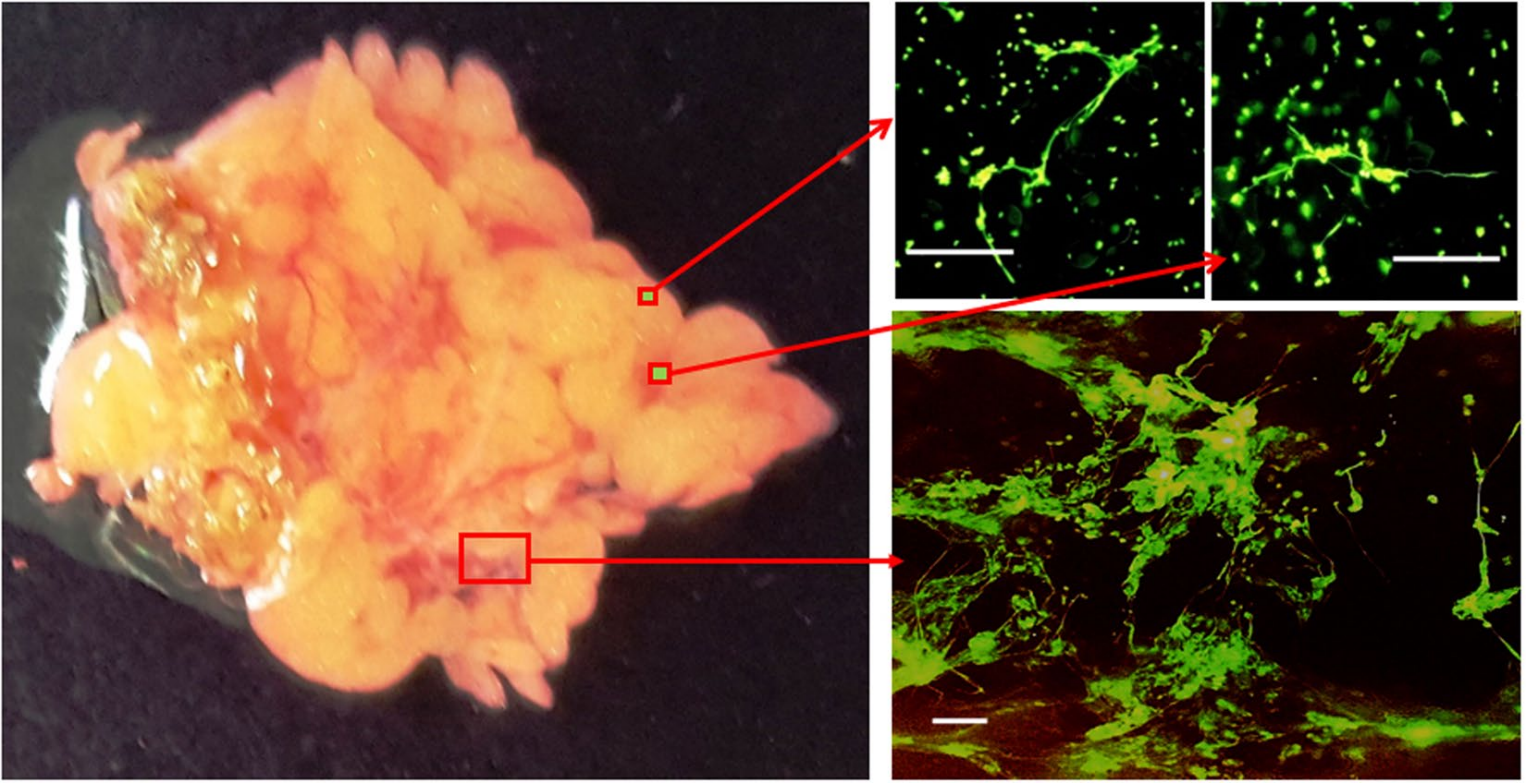
Human omental tissues obtained in gastrectomy were placed in PBS and SYTOX green was added at 50 nM. Then, the nucleic acid components on the mesothelial surface were observed in various magnification using fluorescein stereoscope (Nikon, Kanagawa, JAPAN). Figures of a representative sample among 5 different cases were expressed. White bars show 100 μm.

Finally, we obtained omental tissues from the patients who received radical gastrectomy, and observed the surface structure with the addition of SYTOX green under the fluorescein stereoscope. As shown in Fig. 6, besides the round nucleus probably of the dead cells, many threadlike structures were observed on omental surface. This suggests that many NETs are actually present on human peritoneum after abdominal surgery.

## Discussion

In this study, we hypothesise that the alteration of intraabdominal immunological microenvironment might have important roles on the development of postoperative peritoneal recurrence and examined the characteristics of immune cells contained in the peritoneal lavages after abdominal surgery. Indeed, we found a large number of LDN exist in the postoperative lavages. The LDN contained multilobular nucleus and multiple vacuoles. Their morphology was clearly differennt form that of inmature type LDN which are frequently observed in circulating blood in lupus patients^22,23^. Moreover, flowcytometric analysis revealed that the LDN highly expressed activation markes with reduced L-selectin and IL-8 receptors. Those findings are suggestive that the low density of those LDN may be the results of degranulation. This appears to be reasonable because the LDN in postoperative lavages, whether they are direct exudates or derived from intraoperative bleeding, are supposed to stay on peritoneum for long time and to be subjected to various stimuli.

The LDN produce massive threadlike extracellular DNAs in short term culture without any additional stimuli. The charecteristic SYTOX staining was confirmed as NETs by immunohistochemistry. We have examined the NETs formation in various culture conditions and found that NETs can be detected on various substrates and even without serum, although the amount and time course of NET formation were somehow altered. Previous studies have demonstrated that neutrophils purified from circulating blood of lupus patients are prone to generate NETs, and LDN, in particular, have a markedly enhanced capacity to generate NETs^17,32^. Our results is consistent with those results and suggest that the peritoneal LDN is also highly primed to produce NETs.

Interestingly, human gastric cancer cells efficiently attached to the NETs after a short term co-incubation, which was totally abrogated by the pretreatment by DNAse. Previous studies have already reported the same results using enriched NETs substrates derived from circulating neutrophils, although using different assay protocols^26,27^. Futhermotre, Park *et al*. have recently shown that metastatic breast cancer cells stimulate neutrophils to form NETs which enthanced tumor cell growth in target organ^33^. From these facts, it is supposed that NETs may play crucial role in hematogeneous metastasis. Our data are in line with theirs and suggest the same senario in peritoneal metastasis.

More recently, it has been shown that myeloid leukemea cell, K562^34^ and lung carcinoma cell, A549^35^ showed β1 and β3 integrin dependent adhesion to NETs. Indeed, there are previous *in vivo* studies suggesting that those integrins mediate initial attachment of tumor cells on murine peritoneum^36–38^. From our results, however, it is supposed that the adhesion of those tumor cells to murine peritoneum may be partially dependent on NETs.

In our observation, tumor cells attached to NETs did not die but continued to proliferate in culture. This is a sharp contrast from bacteria which are mostly killed by NETs. This is supposed to be caused by the resistance of tumor cells to the anti-microbial components on NETs. This is not surprising but rather reasonable, because neutrophil elastase, one of the main components of NETs has been reported to enhance tumor cell proliferation^39–41^ and invasion^42,43^ via both direct and indirect manners. Also, Cathepsin G has been reported to induce tumor cell aggregation which is beneficial for metastasis formation^44^. Furthermore, NETs have been reported to highly express neutrophil derived matrix metalloproteinase (MMP)-9, which plays critial roles on degradation of extracellular matrix to facilitate tumor invasion^45^. In fact, enhancement of migration or invasion by NETs substrate has been reported^26,27,33^, although not detected with simple co-culture with “Netted” neutrophil in our experimental system. Taken these facts into consideration, NETs are supposed to provide a favorable microenvironment for the survival of trapped tumor cells.

In our *in vivo* experiments, co-transfer of the peritoneal LDN strongly increased metastatic nodules of MKN45 on murine peritoneum which was dramatically abrogated with the following DNAse treatment. We confirmed that addition of DNAse had negligble effects on tumor cell growth *in vitro* (data not shown). NDN also produced NETs which can trap MKN45 *in vitro* although the amount was much less than LDN. Moreover, co-transfer of NDN slightly, but not significantly, increased metastsis *in vivo*. Together together, this strongly suggests that the presence of NETs derived from exudative neutrophils mediate the adhesion of tumor cells and promote peritoneal metastasis.

In literature search, we found that Van den Tol *et al*. showed that neutrophils of posoperative lavages had crucial role to induce intraabdominal tumor recurrence using rat peritoneal trauma model, although the mechanisms were not examined in detail^46^. Their results are totally consistent with our *in vivo* data and raises a possibility that NETs may be critically involved in their system. In summary, NETs on peritoneum after abdominal surgery gather the disseminated tumor cells and provide a favorable microenvironment for the survival of the cells, and thus disruption of the NETs on peritoneal surface may be clinically useful to prevent postoperative peritoneal recurrence. Probably, the simplest way is to use DNAse in peritoneal washings. It is previously reported that extensive intraoperative peritoneal lavages (EIPL) using 10 liters of saline can effectively suppress the peritoneal recurrence in patients who received curative gastrectomy for advanced gastric cancer, presumably because of the drastic reduction of intraperitoneal cancer cells^47^. However, since NETs as well as LDN should be mostly washed away by the EIPL maneuver, the remarkable clinical effects might be, at least in part, attributed to the depletion of NETs on peritoneum. Therefore, addition of appropriate amount of DNase in washing solution may produce similar clinical effects with much less efforts. Although further *in vivo* study is necessary using orthotopoic metastasis model in syngeneic system, this strategy may be examined in future clinical trials.

## Materials and Methods

### Cells and reagents

Peritoneal cells were obtained from patients who received gastrectomy for gastric cancer during the surgery in Departments of Gastrointestinal Surgery, Tokyo University and Department of digestive Surgery, Jichi Medical University. Informed written consent was obtained from all patients. In short, abdominal cavity was washed with 200 ml and 1000 ml of normal saline just after laparotomy and before wound closure, respectively, and 100 ml of the lavages were collected. After the centrifugation of the peritoneal lavages at 1500 rpm for 15 min, the pellets were resuspended in PBS + 0.02% EDTA and overlaid on Ficoll-Hypaque solution (Pharmacia Biotech, Piscataway, NJ). After centrifugation at 3000 rpm for 15 min, the intermediate and bottom layers were taken and washed twice with PBS + 0.02% EDTA. Granulocyte contained in the former or latter layer was considered as low density neutrophils (LDN) and high density neutrophils (HDN). In selected experiments, the LDN was purified with MACS method using anti-CD66b microbeads as manufacturers’ recommendation (Miltenyi Biotec, Bergisch Gladbach, Germany). Usually, positively selected fraction contains more than 97% LDN. SYTOX green nucleic acid stain and DNAse I were purchased from Thermo Fisher Scientific (Waltham, MA) and PKH26 was from Sigma-Aldrich (St Louis, MO).

This study was carried out in accordance with the Declaration of Helsinki and was approved by the Institutional Review Boards of the University of Tokyo (Approval number: 10034) and Jichi Medical University (Approval number: RIN-A-15-163).

### Cell lines

The human gastric cancer cell line MKN45 and NUGC-4 was obtained from Riken (Tukuba JAPAN) in June 2010, and OCUM-1, authenticated using genomic profiling (IDEXX Radil Cell Check), was obtained from Dr M. Yashiro (Osaka City University) in January 2011. The cells were maintained in Dulbecco’s Modified Eagle Medium (DMEM) supplemented with 10% fetal bovine serum (FBS) (Sigma, St. Louis, MO), 100 units/ml penicillin and 100 mg/ml streptomycin (Life Technologies, Inc., Grand Island, NY) in our laboratory and stored in liquid nitrogen to ensure that cells used for experiments were passaged for fewer than 6 months. No further genomic authentication was performed but cell lines were tested biannually for identity by appearance and growth curve analysis and validated to be mycoplasma free.

### Morphological observation

The postoperative peritoneal cells which consisted mainly of LDN was placed on poly-l-lysine coated slides and stained with Giemsa solution (Wako chemical, Tokyo Japan), and nuclear morphology was observed by microscopy. Also, the cells were plated on coverslips, fixed in 2.5% glutaraldehyde and post-fixed by repeated incubations with 1% osmium tetroxide/1% tannic acid. Then, the samples were dehydrated by using a graded series of ethanol and propylene oxide, after which the samples were observed by using a transmission electron microscope (H7600; Hitachi, Tokyo, Japan).

### Flow cytometry

For immunostaining, 1 × 106 cells were suspended in 100 μl of PBS + 0.02% EDTA, incubated with 10 μl of Fc-blocker for 20 min and then incubated with FITC- conjugated CD66b and PE-conjugated each mAb to CD11b, CD15, CD62L, CD63, and Interleikin-8 receptor A and B as well mouse IgG (Becton-Dickinson, San Jose, CA) for 30 min in 4 °C as per the manufacturer’s recommendation. Then, the alive granulocytes were gated as CD66b(+) 7-AAD(−) area and expression of each antigen was analyzed in the gated area using FACS-Caliber (Becton-Dickinson, San-Jose, CA).

### Fluorescence microscopy

The peritoneal LDN (5 × 106) suspended in 1 ml DMEM supplemented with 10% FCS were cultured on poly-l-lysine coated 6 well plate. After indicated periods, SYTOX green was added at the concentration of 50 nM, and the plate was observed with a fluorescence stereomicroscope (BZ8000, Keyence, Osaka, Japan). In quantification of NETs, the areas which showed characteristic strand staining for SYTOX green were encircled in 3 randomly selected fields and quantified using an imageJ software (NIH, Bethesda, MD).

### Fluorescein stereoscope

Human omental tissues were obtained from patients who received gastrectomy for gastric cancer. Immediately after the resection of stomach, a small part of omental tissue was removed from the specimen, gently washed in PBS and transferred into the dish in PBS, and SYTOX green was added at the final concentration of 50 nM. Then, the specimen was observed under fluorescein stereoscope (Nikon, Kanagawa, JAPAN) using the appropriate wavelength filter for FITC.

### Adhesion assay and proliferation of adhesive tumor cells

MKN45, OCUM-1 or NUGC-4 were stained red by PKH26 as manufacturers’ recommendation. The peritoneal LDN (5 × 106) were confluently cultured on poly-l lysine coated 6 well plate for 2 hours. After the washing, the tumor cells (1 × 106) resuspended in 1 ml DMEM were added and incubated for 5 min for all the tumor cells to contact the LDG monolayer. Then, the wells were gently washed for 3 times and attached tumor cells were counted using the specific filter for PHK26 under the fluorescein microscope. In some experiments, DNAse I were added on the LDN culture at the final concentration of 100 u/ml and incubated for 5 min, washed and then tumor cells were added. For visualization of NETs, SYTOX green was added at the concentration of 50 nM on the washed plate and observed under the fluorescein microscope with different optical wavelength filter for FITC.

In some experiments, the wells were washed twice again and continued the co-culture in 10%FCS + DMEM, and the outcome of the attached tumor cells was observed with fluorescein microscope or time lapsed video analysis sing a Biostudio system (Nikon Engineering, Kanagawa, Japan) and Image Viewer software (Nikon Engineering, Kanagawa, Japan) as described previously (32).

### *In vivo* experiments

Peritoneal metastasis of human gastric cancer cells in nude mice was examined as described previously (33). In brief, four-week-old specific-pathogen-free conditioned female BALB/c nude mice were purchased from Clea Japan Inc. (Fijinomiya, Japan), and maintained in a temperature controlled, light cycled room. At 5 weeks after birth, the mice were intraperitoneally (IP) inoculated with MKN45 cells (1 × 105~2.5 × 105) suspended in 1 ml Hanks balanced solution (HBSS). In the case of co-transfer experiments, 1 × 107 LDN suspended in HBSS were mixed just before IP inoculation. After 4 weeks, the mice were sacrificed and metastatic nodules on mesenteric peritoneum were counted. In some experiments, DNase I suspended in HBSS at the concentration of 1000 u/ml were IP administrated just after tumor cell injection.

### Statistical analysis

The results were statistically examined by paired Student’s t tests or Wilcoxon’s test when appropriate. Results are given as means, and differences with P < 0.05 were considered to be significant.

### Data availability

All the datasets generated during and/or analyzed during the current study are available from the corresponding author on reasonable request.

## Electronic supplementary material

Video 1

Video 2

Video 3

Supplememtary information
