# Supplementary material for: Low density neutrophils (LDN) in postoperative abdominal cavity assist the peritoneal recurrence through the production of neutrophil extracellular traps (NETs)
Source: Sci Rep. 2018 Jan 12;8:632. doi: 10.1038/s41598-017-19091-2 (PMC5766579; doi:10.1038/s41598-017-19091-2)
Supplement: Supplementary file 4 — Supplememtary information [file 41598_2017_19091_MOESM4_ESM.pdf]

Low density neutrophils (LDN) in postoperative abdominal cavity assist the peritoneal recurrence through the production of neutrophil extracellular traps (NETs)

Running title: NETs and peritoneal metastasis

Rihito Kanamaru[1), Hideyuki Ozawa[1), Hideyo Miyato[1), Shiro Matsumoto[1), Hidenori Haruta[1), Kentaro Kurashina[1), Shin Saito[1), Yoshinori Hosoya[1), Hironori Yamaguchi[1), Hiroharu Yamashita[2), Yasuyuki Seto[2), Alan Kawarai Lefor[1), Naohiro Sata[1), and Joji Kitayama[1)
1) Department of Gastrointestinal Surgery, Jichi Medical University
2) Department of Gastrointestinal Surgery, The University of Tokyo

Corresponding author:
Joji Kitayama, MD
Department of Gastrointestinal Surgery, Jichi Medical University
Yakushiji 3311-1, Shimotsuke, Tochigi
329-0498 JAPAN
Tel:81-285-58-8941
FAX:81-285-44-6811
e-mail: kitayama-jichi.ac.jp

Supplement Figure 1

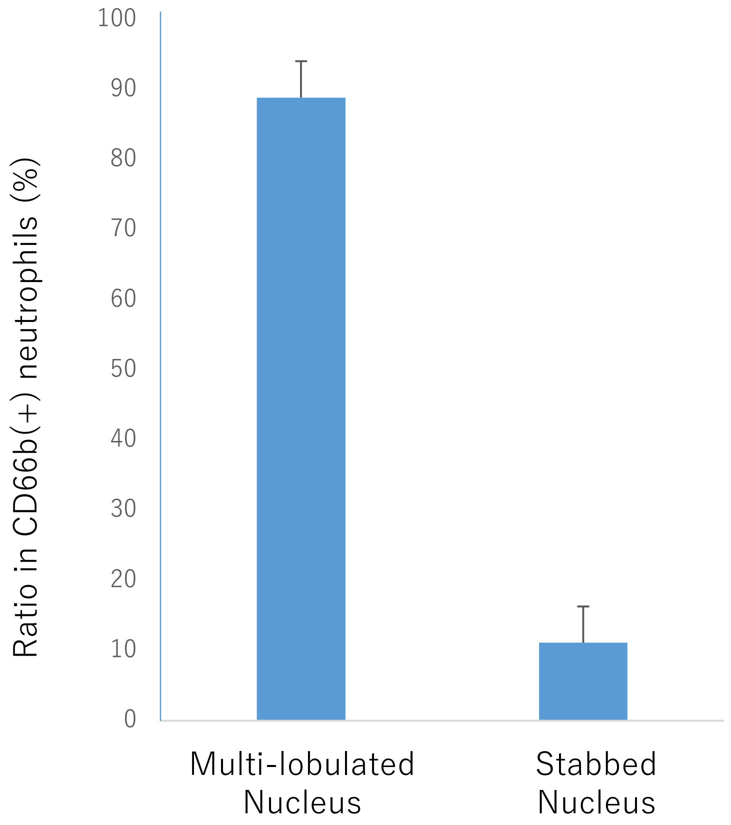

Postoperative peritoneal lavages were obtained in 3 different patients and CD66b(+) cells were purified after Ficoll-Hypaque centrifugation and stained by Giemsa Method. Shape of nucleus were examined in low magnification and calculated the ratio of cells with lobulated and stabbed nucleus. Mean ± SD were expressed.

Supplement Figure 2

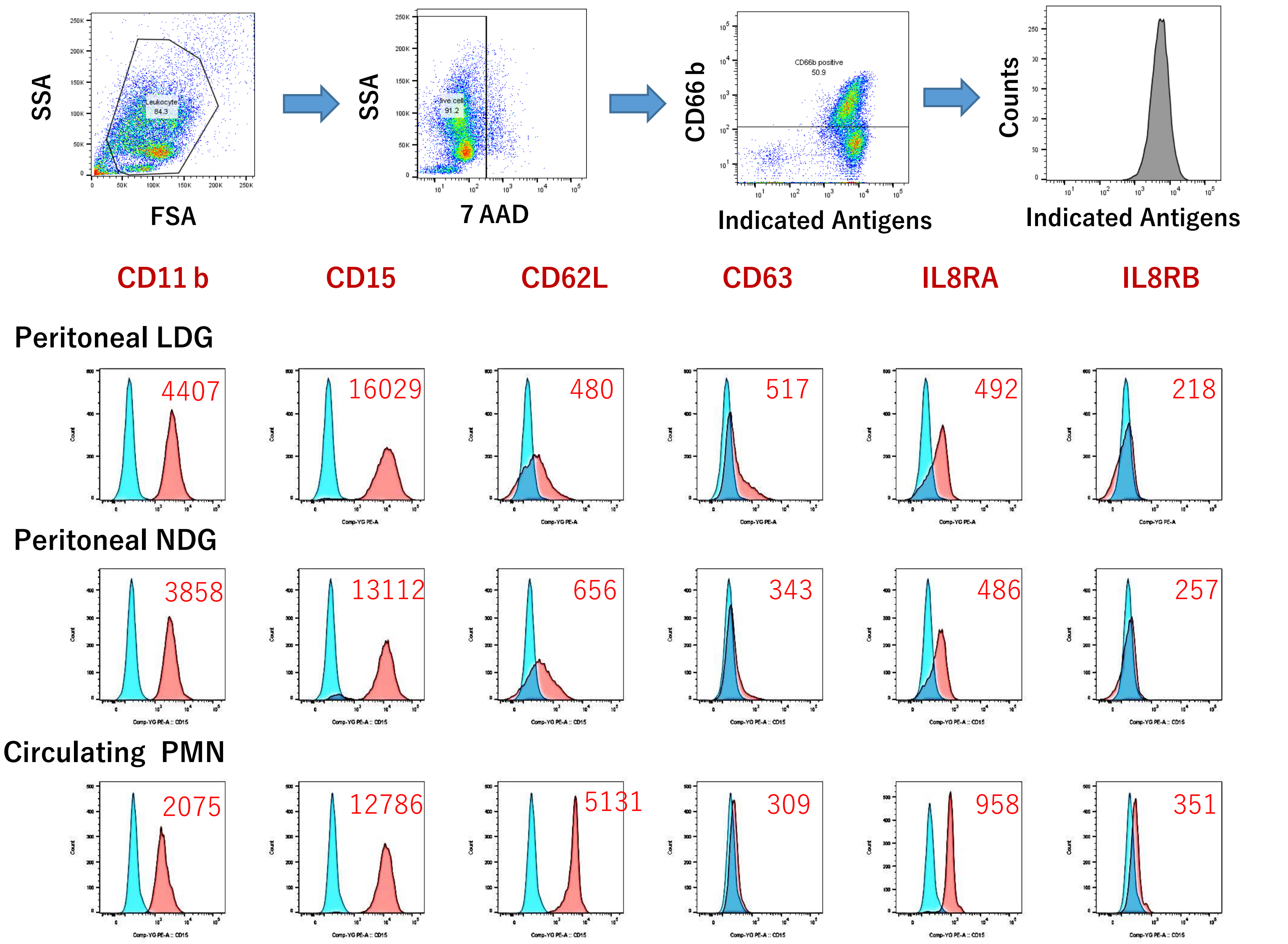

Antigen expression pattern of LDN and HDN in postoperative lavage and circulating PMN in a representative patient. Upper panel shows the gating strategy. First, leukocyte range was gated, then 7AAD positive dead cells were removed, and CD66b-positive neutrophils were gated. The expression of each antigen on live neutrophils in a representative patient were expressed by histogram. Red and blue figure show the fluorescein staining with mAbs to specific antigens and control IgG, respectively, and number show the mean fluorescein channel (MFC).

Supplement Figure 3

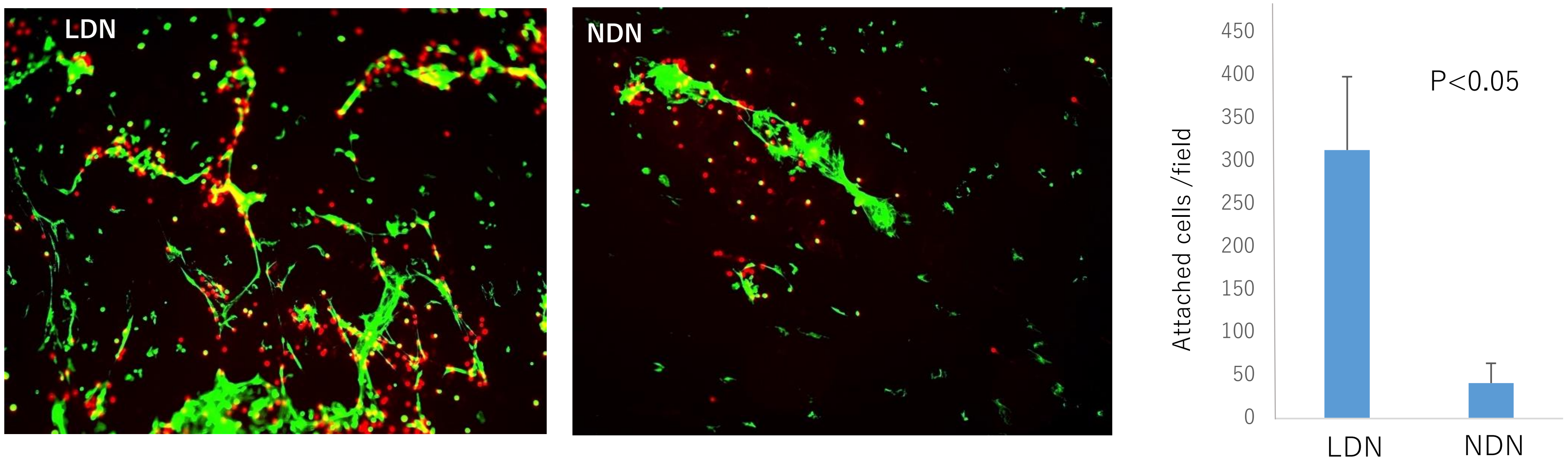

MKN adhesion to LDN (left) and NDN (right). LDN or NDN (5×10^{6}) from a same patient were cultured on poly-L-lysine coated 6 well plate for 2 hours, and PKH26 stained MKN45 (1×10^{6}) cells suspended in 1 ml DMEM were added and incubated for 5 min. After gentle washing, SYTOX green was added and NET and tumor cells were visualized under the optical wavelength filter for FITC and PE, respectively, and superimposed. The number of attached MKN45 were counted in 3 different fields and mean ± SD were expressed.

.

Supplement Figure 4

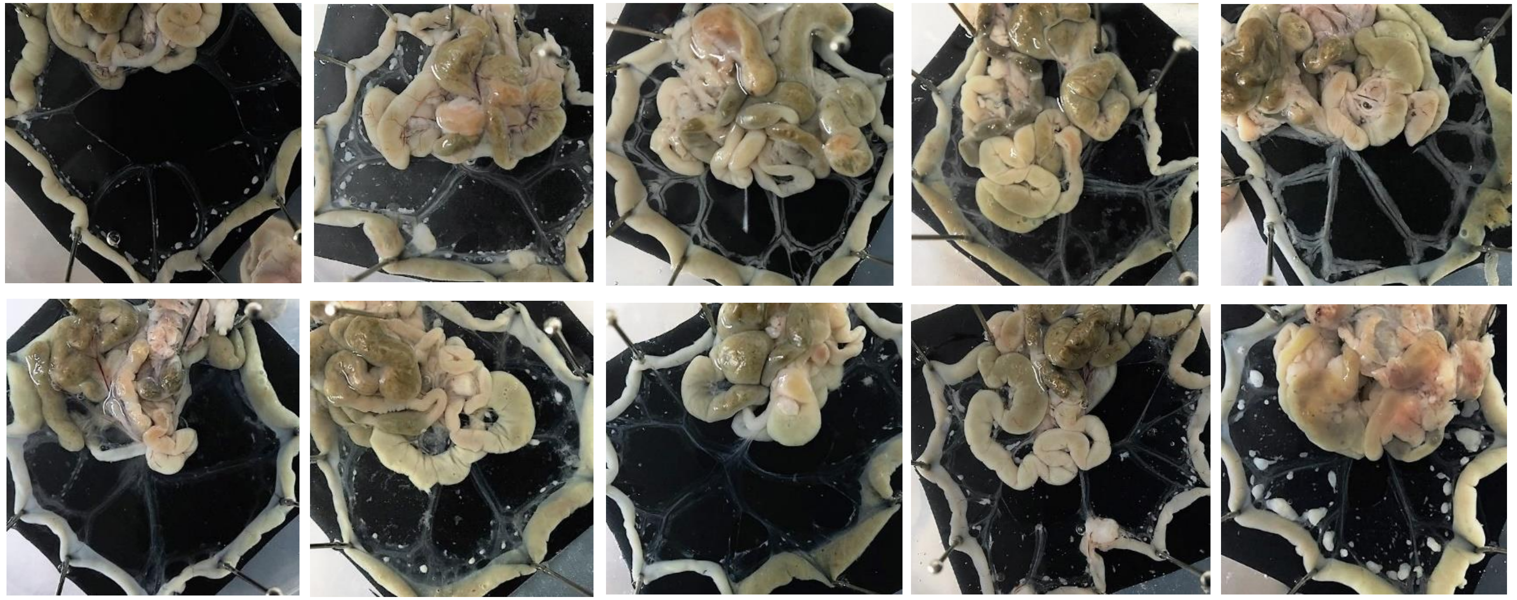

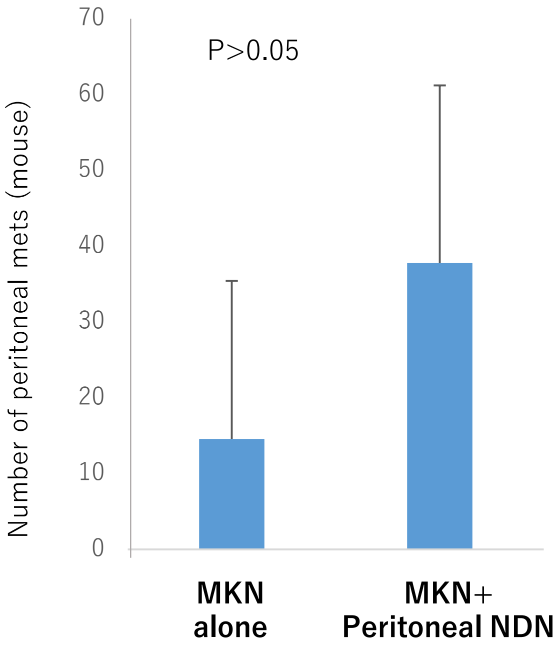

MKN45 ($1x10^5$) with (lower) or without (upper) purified NDN ($1x10^7$) suspended in 1 ml HBSS were intraperitoneally injected into the balb/c mice. After 4 weeks, the mice were sacrificed and the number of macroscopically detectable metastatic nodules on mesentery were counted. Mean±SD in 5 mice in a representative experiment were shown in right panel. Although co-transfer of NDN tended to increase metastatic nodules, the difference was not statistically significant.

Supplement Video legend.

LDN or NDN ($5x10^6$) were cultured on poly-L-lysine coated 6 well plate for 2 hours, and MKN45 (1x106) were added on them. After 5 min incubation, the well was gently washed and SYTOX green was added to detect NETs. Then, the wells were washed twice and continued the co-culture in 10%FCS+DMEM and the outcome of the attached MKN45 was observed by time lapsed video analysis using a Biostudio system (Nikon Engineering, Kanagawa, Japan).
